# *In silico* performance of a targeted enriched metagenomics approach to infer *Mycoplasma bovis* strains in milk

**DOI:** 10.3389/fvets.2026.1770245

**Published:** 2026-03-18

**Authors:** Marit M. Biesheuvel, Herman W. Barkema, Paul S. Morley, Lee J. Pinnell, Enrique Doster, Robert Valeris-Chacin

**Affiliations:** 1 Faculty of Veterinary Medicine, University of Calgary, Calgary, AB, Canada; 2 VERO Program, College of Veterinary Medicine and Biomedical Sciences, Texas A&M University, Canyon, TX, United States

**Keywords:** dairy, metagenomics, milk, *Mycoplasma bovis*, strain detection, targeted-enrichment

## Abstract

Strain variation plays a key role in the microbial epidemiology of *Mycoplasma bovis*, yet its true diversity remains incompletely characterized, partly due to limitations of culture-based methods. This study evaluated the *in silico* suitability of a targeted enrichment (TE) shotgun sequencing approach to detect and classify *M. bovis* strains in milk metagenomic samples. As a proof of concept, the accuracy of this approach was assessed using milk-derived *M. bovis* strains. A total of 620 *M. bovis* whole-genome sequences were downloaded from NCBI, of which 162 (26.1%) originated from milk samples. Genomes were grouped into Genomically Clustered Sequence Variants (GSVs) using MashTree and TreeCluster to enable strain-level classification. To simulate TE sequencing data, genomes from different milk-associated GSVs were randomly selected and fragmented *in silico* into 150-bp reads. Mock milk samples were generated by sampling reads with replacement from these genomes. Sequencing depth was modeled using a Poisson distribution, while mixed-strain DNA samples were simulated by including 1, 3, 6, or 9 GSVs per sample. Enrichment proportions were set at 0.3, 0.5, 0.7, and 0.9. Two classification tools, Kraken2 and Themisto/mSWEEP, were evaluated for their ability to detect and classify the simulated TE reads. Themisto/mSWEEP consistently outperformed Kraken2, achieving an average read classification accuracy of 84.9% compared with 1.4% for Kraken2. Sensitivity for Themisto/mSWEEP was 100% with a single spiked GSV and declined slightly to 97.0% with nine GSVs, whereas Kraken2 achieved sensitivities of only 17.3% and 4.7%, respectively. Positive predictive value (PPV) showed a similar pattern: 98% for Themisto/mSWEEP vs. 4.7% for Kraken2 with a single GSV, and 65.5% vs. 10% with nine GSVs. While Kraken2's PPV increased slightly with additional GSVs, Themisto/mSWEEP's PPV decreased. Both methods maintained high specificity and negative predictive value (>91%) across all scenarios. Enrichment proportion had no measurable effect on performance. Overall, Themisto/mSWEEP demonstrated superior accuracy for GSV-level identification of *M. bovis* strains. Enrichment to at least 30% of total reads was sufficient to recover strain-level data. Further work is needed to assess the biological relevance and practical applications of these genomic clusters.

## 1 Introduction

*Mycoplasma bovis* infections are widespread in dairy cattle globally (1, 2), presenting with diverse clinical signs including mastitis, arthritis, pneumonia, otitis media (3), while some cattle remain subclinical carriers (4). Despite efforts on herd level and national level, prevalence has not declined, highlighting ongoing challenges in control and eradication. Genomic diversity of *M. bovis* remains understudied (5), though it may influence outbreak dynamics and disease progression. Fox (6) documented new *M. bovis* strains emerging after clinical outbreaks, closely resembling the initial strain but not causing disease. Cases of arthritis originating from pneumonia or mastitis suggest possible internal somatic spread (6), though underlying mechanisms remain unclear. Transmission dynamics are equally variable: a single cow can infect up to 80 other susceptible cows during an outbreak, while transmission rates remain slow in other cases, with no clear links to management practices (7). These examples emphasize the importance of further exploring *M. bovis* strain diversity, which is increasingly recognized as a key factor influencing the pathogen's microbial epidemiology (8–11). However, culture-based techniques have limitations in elucidating these complexities.

Over the past decade, high-throughput sequencing has advanced pathogen research, providing insights into origin, transmission, virulence and antimicrobial resistance (12, 13). However, detecting genetic variations as small as 0.1%−5% nucleotide differences remain challenging. Traditional approaches rely on DNA extraction after bacteriological culturing, introducing numerous biases, including low sensitivity of *M. bovis* culture procedures, and long turnaround time due to the fastidious and slow-growing nature (14). While culture-free methods allow for direct DNA extraction and sequencing, they are still relatively new, complex and resource-intenstive, making them difficult to implement (15).

Targeted enrichment (TE) methodologies can address some of these limitations in sequencing *M. bovis* from metagenomic samples. Target-enriched metagenomics selectively capture specific genomic regions, allowing for the sequencing of low-abundance gene sequences and identifying genetic variations (16–18). This is especially valuable for pathogens like *M. bovis*, which is usually present at low abundance in metagenomic samples, such as milk, where host DNA dominance and low pathogen copy number (< 1%) can make detection difficult (19). By reducing host DNA interference, targeted enrichment enables deeper sequencing coverage of the pathogen's genome and facilitates the detection of rare variants that could potentially play a role in outbreak dynamics or antimicrobial resistance development.

Despite these advancements, strain identification remains a challenge (20). Traditional alignment-based methods, which rely on identifying single-nucleotide variant (SNV) can be computationally intensive and time-consuming. In contrast, tools like MashTree (21) and TreeCluster (22) offer a more efficient alternative by calculating genomic distances using MinHash sketches and grouping genomes based on their phylogenetic relationships, rather than relying on single nucleotide polymorphism (SNP) or SNV calling.

Bioinformatic tools like Kraken2 (23), Themisto (24), and mSWEEP (25) provide efficient ways for assessing mixed-strain DNA samples, which have gained increasing importance in recent years. It is now recognized that infections can involve multiple strains of a single pathogen (26). Mixed infections can complicate diagnosis, treatment, and control measures, increasing the risk of treatment failure (20, 27). However, discrimination amongst these strains is not fully understood and accurate thresholds for clustering have not been established.

Although the potentiality to obtain strain-level classification of *M. bovis* via targeted enriched metagenomics exists, its performance in metagenomic samples has not yet been fully explored. Therefore, the objectives of this study were to assess, *in silico*, the feasibility and performance of a TE shotgun sequencing approach for *M. bovis* strain detection in milk metagenomes, and to benchmark Themisto/mSWEEP against Kraken2 under controlled simulated scenarios.

## 2 Materials and methods

### 2.1 Study overview

**Figure 1:**
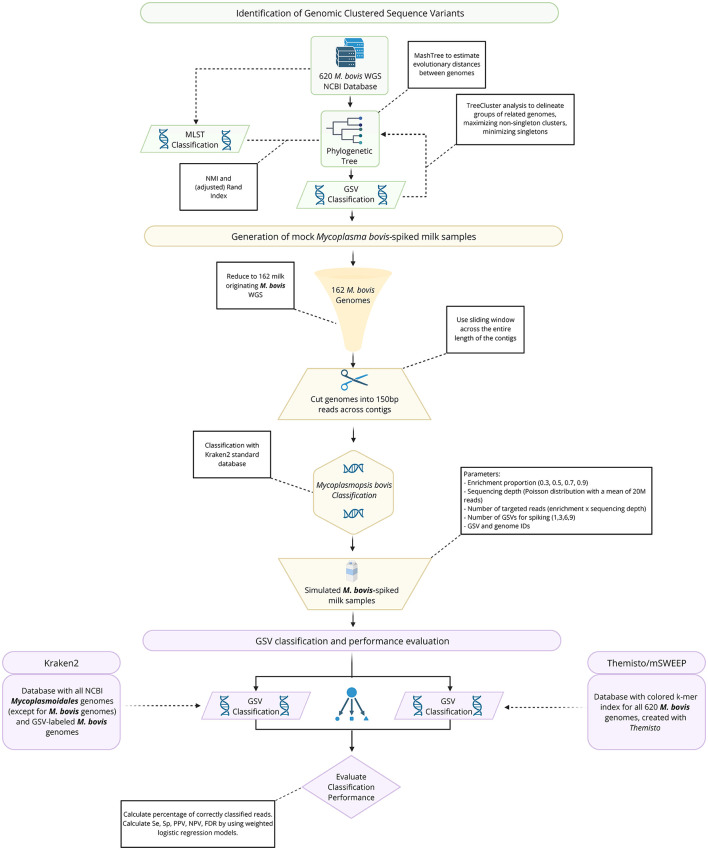
Flowchart depicting the study overview.

This study aimed to evaluate, *in silico*, the feasibility and performance of a TE shotgun sequencing approach for detecting *M. bovis* strains in milk samples, and to compare Themisto/mSWEEP with Kraken2 under controlled simulated scenarios (Figure 1). Background reads from metagenomic shotgun sequencing (i.e., milk microbiome and host) were not included, as the bioinformatic approach used in this study inherently removes reads mapping to host genome (cattle) and non-*M. bovis* reads using Kraken2. Including these background reads would have significantly increased computational demands without offering additional insights (16). The simulation focused on two key parameters: the enrichment proportion (ranging from 30 to 90%) and the number of distinct *M. bovis* strains (ranging from 1 to 9). To address the lack of standardized methods for strain classification, we clustered publicly available *M. bovis* genomes based on their phylogenetic relationships and refer to these clusters as Genomically Clustered Sequence Variants (GSVs) (16).

### 2.2 Genomes

All *M. bovis* whole genome sequences (WGS) from *Bos taurus*, along with their associated metadata, were retrieved from the National Center for Biotechnology Information (NCBI) database (28) on October 23, 2023 (*n* = 620, Supplementary Table 1). This comprehensive dataset comprises sequences obtained from both the RefSeq and GenBank databases. For each genome, the metadata included information on country of origin and sample location on the animal, which were categorized as originating from milk, non-milk sources, or source information not available.

### 2.3 Classification of genomes into genomically clustered sequence variants

All *M. bovis* WGS in FASTA format were used as input for MashTree version 2.0 (21) to calculate the Mash distances (29). These distances are derived from MinHash sketches, which provide a compressed representation of the genomic sequences (29). The Mash distance, based on the Jaccard dissimilarity of shared k-mers, serves as a rapid and scalable approach for estimating evolutionary distances between genomes (29). Finally, MashTree used the neighbor-joining algorithm (30) to construct a phylogenetic tree from the pairwise Mash distances.

Following the phylogenetic tree construction with MashTree, genomic clustering was performed using TreeCluster version 1.0.3 (22) to delineate groups of related genomes. TreeCluster applies a hierarchical clustering approach. We evaluated 14 different clustering methods and 22 thresholds for each one (Supplementary Figure 1). The optimal clustering method and threshold combination, which maximized the number of non-singleton clusters while minimizing the number of singletons, was selected to define genomic clusters of *M. bovis* genomes. These clusters, referred to as genomically clustered sequence variants (GSVs), provided a framework for identifying and characterizing distinct genomic variations within *M. bovis* population. All GSVs were each assigned a unique identifier. Additionally, the MLST classification (both the current and legacy schemes) for the *M. bovis* genomes was obtained uploading each genome to https://pubmlst.org (31). The similarity between the GSV and MLST classifications was estimated via Normalized Mutual Information (NMI) (32), Rand index, and adjusted Rand index (33) from the R package igraph version 2.1.4, and the variation of information (VI) (34) from the R package mcclust version 1.0.1.

MashTree generated a phylogenetic tree in Newick format, which was then visualized in R 4.4.1 using the “ggtree” package version 3.16.0 (35). To enhance the visualization and provide additional context, sample type (milk, non-milk, and missing) and GSV identifier based on TreeCluster analysis were incorporated for each genome. This integration was achieved using the “gheatmap” function from the ggtree package.

### 2.4 Generation of mock *Mycoplasma bovis*-spiked milk samples

To create simulated *M. bovis*-spiked milk samples, only genomes of *M. bovis* isolated from milk samples (*n* = 162) were utilized. Initially, each genome was bioinformatically split into 150-base pair reads using a sliding window across the entire length of the contigs (in-house script, “Cutting_genomes_final2.R” available in the GitHub repository). Then, these reads underwent classification using the Kraken2 standard database, retaining only reads classified as *Mycoplasmopsis bovis* or lower. This step simulates the removal of reads mapping to the host genome (cattle) and non-*M. bovis* reads present in real milk metagenomic samples.

Subsequently, a parameter file was generated in R 4.4.1 with the different scenarios and parameter settings for the simulation (see “Gridfile_parameters.R” and “grid_parameters_new.csv” in the GitHub repository). This parameter file explicitly stated the enrichment proportion, sequencing depth, number of targeted reads, number of GSVs for spiking, the GSV identifiers and the genome IDs to be sampled within the GSVs for each iteration of the simulation. The enrichment proportion encompassed values of 0.3, 0.5, 0.7, and 0.9 to mimic different levels of targeted enrichment. The sequencing depth was randomly drawn from a Poisson distribution with a mean of 20,000,000 reads (36, 37). Subsequently, this sequencing depth was multiplied by the enrichment proportion to determine the targeted number of reads to be sampled. The aim was to model various scenarios of *M. bovis* mixed-strain DNA samples, with the number of GSVs spiking a sample ranging from 1, 3, 6, and 9. The specific GSVs were randomly sampled without replacement from the total list of GSVs established by TreeCluster to ensure diversity in the genomic variants represented in the spiked samples. Then, since multiple genomes could belong to the same GSV, genomes for a specific iteration were randomly sampled without replacement within the specified GSV, and the genome IDs were added to the parameter file. For each combination of parameter settings of enrichment proportion and number of GSVs used for spiking, 1,000 iterations were performed, resulting in a total of 16,000 iterations.

During each iteration, genomes specified in the parameter file were concatenated when more than 1 GSV was involved. Next, the “sample” function in Bash (38) was employed to sample with replacement to obtain the targeted number of reads from this concatenated file. After generating the mock *M. bovis-*spiked milk samples, the reads were classified using two approaches (Kraken2 with a custom database and the Themisto/mSWEEP tool) to determine the GSVs present in each mock sample (see files “simulation_kraken2_batch1.txt” and “simulation_msweep_batch1.txt” in the GitHub repository).

### 2.5 Kraken2 with a custom database

A custom Kraken2 database was created to classify the sampled reads, following guidelines outlined in the Kraken2 manual, using the default parameters, which include a k-mer length of 35 base pairs and a minimizer length of 31 base pairs (23). All *Mycoplasmoidales* genomes (except for *M. bovis* genomes) were downloaded from NCBI on December 2, 2023. The downloaded *Mycoplasmoidales* genomes in addition to the GSV-labeled *M. bovis* genomes were incorporated into the database using the kraken2-build tool. The custom database was then used to classify the reads in each mock sample.

### 2.6 Themisto/mSWEEP tool

Themisto, a pseudoalignment tool for bacterial genomes (24), was employed as an alternative method to classify the reads from the mock *M. bovis*-spiked milk samples. First, all 620 *M. bovis* genomes were utilized to create a colored k-mer index via Themisto, with its default k-mer length of 31 base pairs. Pseudoalignment of the mock *M. bovis*-spiked milk sample reads to the index using k-mer matching was then performed via Themisto. Finally, mSWEEP (25) was employed to robustly estimate the GSV relative abundance in the mock *M. bovis*-spiked milk samples by analyzing the pseudoalignment output and the clustering of the *M. bovis* genomes into GSVs.

### 2.7 Estimating performance of target enrichment

For both classification methods, the percentage of reads classified as the correct GSV was estimated. For iterations with multiple GSVs, the percentages classified as each GSV were summed to calculate the total percentage for that iteration. The unit of analysis throughout was the GSV.

To evaluate performance, sensitivity (Se), specificity (Sp), positive predictive value (PPV), negative predictive value (NPV) and false-discovery rate (FDR) were calculated. First, only GSVs with ≥1% of reads classified as the specific GSV (Kraken2) or ≥1% relative abundance (Themisto/mSWEEP) were selected. As a sensitivity analysis, thresholds of ≥5 and ≥10% were also assessed to evaluate the robustness of the results (Supplementary material). Then, for each iteration the following values were determined: true positives (TP), false-negatives (FN), false-positives (FP) and true negatives (TN). True positives were defined as the number of GSVs correctly identified in both the parameter file and output files. False-negatives were defined as the total number of spiked GSVs (1, 3, 6, or 9) minus the number of matching GSVs (TPs). False-positives were defined as the total number of GSVs in the output files minus the number of matching GSVs (TPs), and TNs as the total number of GSVs across all *M. bovis* genomes minus the total number of spiked GSVs (1, 3, 6, or 9) and the FPs. Again, the unit of analysis throughout was the GSV. Se, Sp, PPV, NPV, and FDR were calculated using the following formulas:


$$\begin{array}{r} Se=\;\frac{True\;Positives\;}{True\;Positives+False\;Negatives\;}\;x\;100\;\\ Sp=\;\frac{True\;Negatives\;}{True\;Negatives+False\;Positives\;}\;x\;100\;\\ PPV=\;\frac{True\;positives\;}{True\;Positives+False\;Positives\;}\;x\;100\;\\ NPV=\;\frac{True\;Negatives\;}{True\;Negatives+False\;Negatives\;}\;x\;100\;\\ FDR=\;\frac{False\;Positive\;}{False\;positives+\;True\;positives\;}\;x\;100\; \end{array}$$

### 2.8 Statistical analysis

Weighted logistic regression models were employed to assess the effect of the classification method, the number of spiked GSVs, and the enrichment proportion on the diagnostic test performance indicators (Se, Sp, PPV, NPV, and FDR). Prior to running each model, the dataset was expanded to include four rows per iteration per method, corresponding to FP, FN, TP, and TN.

For Se, the simulation results were the outcome variable, coded as 1 for TP or FP, and 0 for TN or FN. Only rows where the true status was one were included in the analysis (only TP and FN). For PPV, the true status was the outcome variable, with only positive simulation results included in the analysis (only TP and FP). FDR was analyzed similarly to PPV, but with 1—true status as the outcome variable. For Sp, the outcome variable was again based on simulation results but coded as one for TN and FN, and 0 for TP and FP, with only rows where the true status was 0 included in the analysis (only TN and FP). Lastly, for NPV, 1 - true status was used as the outcome, and only negative simulation results were included in the analysis (only FN and TN).

Frequency weights corresponding to FP, FN, TP and TN, depending on the row, were used when running the logistic regression models. All models were built using a manual backwards elimination technique, starting with a three-way interaction between enrichment proportion, number of spiked GSVs, and classification method. Only significant interactions were retained in the final models.

Data cleaning and descriptive statistics were performed in R 4.4.1 (39), and the corresponding scripts can be found in the GitHub repository (“Summary_Kraken2.R” and “Summary_mSWEEP.R”). The statistical analyses were performed using STATA/SE Version 16.1 (40), and the corresponding script can be found in the GitHub repository (“OneModel.do”). Results were considered statistically significant at *P* ≤ 0.05.

### 2.9 Sensitivity analysis

An investigation of the impact of the minimizer length and k-mer length (for Kraken2 and Themisto, respectively) on the performance of the classification methods and the targeted enrichment was conducted. Therefore, two additional databases were created for Kraken2 and Themisto with 20 and 25 minimizer length and k-mer length, respectively. Importantly, even though databases can be built with higher k-mer length than the default in Themisto (31 kmers), that is not recommended for Kraken2, which motivated our selection of lower values to test, which may be useful in computing environment with memory restrictions. The sensitivity analysis was performed randomly selecting iterations from the parameter file used in the main simulation stratifying by spiked GSVs (*n* = 150 iterations per spiked GSV group) from the enrichment proportion of 0.3. The selected iterations were randomly assigned to one minimizer length/k-mer length value (20, 25, and the default 31) for a total of 50 replicates per combination of experimental factors. The sensitivity analysis was similar to the main simulation with the following modification: substitution of the database depending on the assigned minimizer length/k-mer length value and generation of reads simulating Illumina Novaseq error rates from the corresponding *M. bovis* genomes instead of the sliding window approach used in the main simulation. The simulated reads (1 million reads per genome with uniform coverage) were created using InSilicoSeq (41).

## 3 Results

### 3.1 *Mycoplasma bovis* genomes

A total of 620 WGS of *M. bovis* from *Bos taurus* were obtained from the NCBI database. Of these, 162 (26.1%) genomes originated from milk samples, while 358 (57.7%) genomes were derived from non-milk samples. The remaining 100 (16.1%) genomes had unspecified sample types (Supplementary Table 1).

### 3.2 Phylogenetic tree and genomically clustered sequence variants

**Figure 2:**
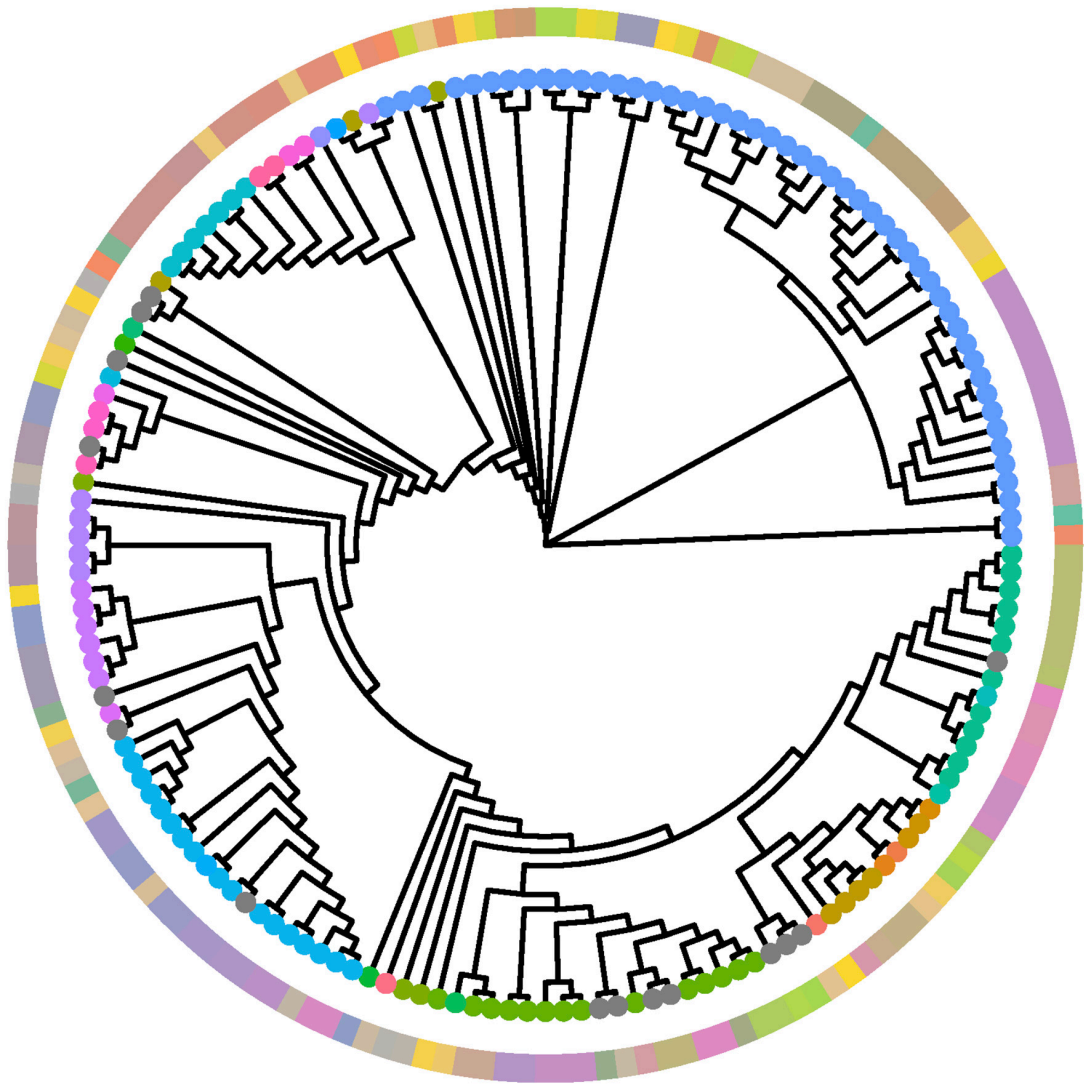
Phylogenetic tree (neighbor-joining method) of 162 *Mycoplasma bovis* whole genome sequences from *Bos taurus* derived from milk. The tips of the tree are color coded according to the MLST classification. The outer ring is color coded by the genomically clustered sequencing variant (GSV) classification according to the average length of the clade with clustering threshold 0.0001.

TreeCluster analysis identified the optimal method for clustering as the average clade approach with a threshold of 0.0001 (Supplementary Figure 1). This method resulted in the identification of 351 clusters, including 168 singletons. When analyzing only the WGS from milk samples, TreeCluster identified a total of 104 clusters, including 43 singletons. *Mycoplasma bovis* genomes derived from milk did not cluster together but were instead intermixed with genomes derived from non-milk and non-specified samples. In addition, within the milk sample clusters, often multiple GSVs were identified (Figure 2). A subset of the *M. bovis* genomes could be unequivocally assigned to a MLST (*n* = 487). A total of 76 MLSTs and 265 GSVs were obtained for those genomes (Supplementary Table 1). The classifications based on GSVs and MLSTs were similar as shown by the values of the NMI and the related VI, 0.74 and 3.24, respectively. A NMI of 0 indicates no mutual information and one perfect correlation, whereas a VI of 0 indicates perfect correlation and no mutual information is shown by the theoretical maximum [in this case, log_(487)_ = 6.19]. Additionally, a high similarity was estimated by the Rand index (0.919). However, most of that similarity seems to be heavily influenced by randomness as the adjusted Rand index was low (0.07). The correspondence between the GSV and MLST classification for isolates from milk samples is shown in Figure 2.

### 3.3 Performance of Kraken2 with a custom database (≥1% of reads classified as the specific GSV)

The average percentage of reads classified correctly as the spiked GSV(s) by Kraken2 was 1.4% [standard deviation (SD) = 2.5%], with a first quartile of 0.44% and third quartile of 1.4%. Mean percentage of correctly classified reads did not vary across different enrichment proportions (lowest = 1.4%, highest = 1.5%) or the number of spiked GSVs (lowest = 1.3%, highest = 1.5%) (Table 1).

**Table 1:** Descriptive statistics, including mean, standard deviation (SD), interquartile range, and total number of iterations (*N*), of the percentage of reads (%) classified as the correct GSV for each simulation parameter (enrichment percentage and the number of spiked GSVs) for Kraken2 and Themisto/mSWEEP.

**Parameter**	***N***	**Mean**	**Median**	**SD**	**IQR**
***Kraken2***					
**Enrichment**					
30%	4,000	1.4	0.7	2.6	0.4–1.4
50%	4,000	1.4	0.7	2.3	0.5–1.3
70%	4,000	1.4	0.7	2.4	0.5–1.4
90%	4,000	1.5	0.7	2.6	0.4–4.5
***N*** **of GSV's**					
1	4,000	1.4	0.5	4.0	0.2–0.8
3	4,000	1.3	0.6	2.1	0.4–0.9
6	4,000	1.4	0.7	1.5	0.5–2.1
9	4,000	1.5	0.9	1.2	0.6–1.9
***Themisto/mSWEEP***					
**Enrichment**					
30%	4,000	85.0	86.2	14.0	73.9–100
50%	4,000	84.8	85.4	14.0	73.8–100
70%	4,000	84.9	85.8	14.1	73.1–100
90%	4,000	84.8	85.1	13.9	73.9–100
***N*** **of GSV's**					
1	4,000	98.8	100	6.3	100–100
3	4,000	90.2	93.6	10.9	83.7–100
6	4,000	78.4	78.7	10.3	71.5–85.8
9	4,000	71.9	71.9	9.4	65.7–78.3

**Figure 3:**
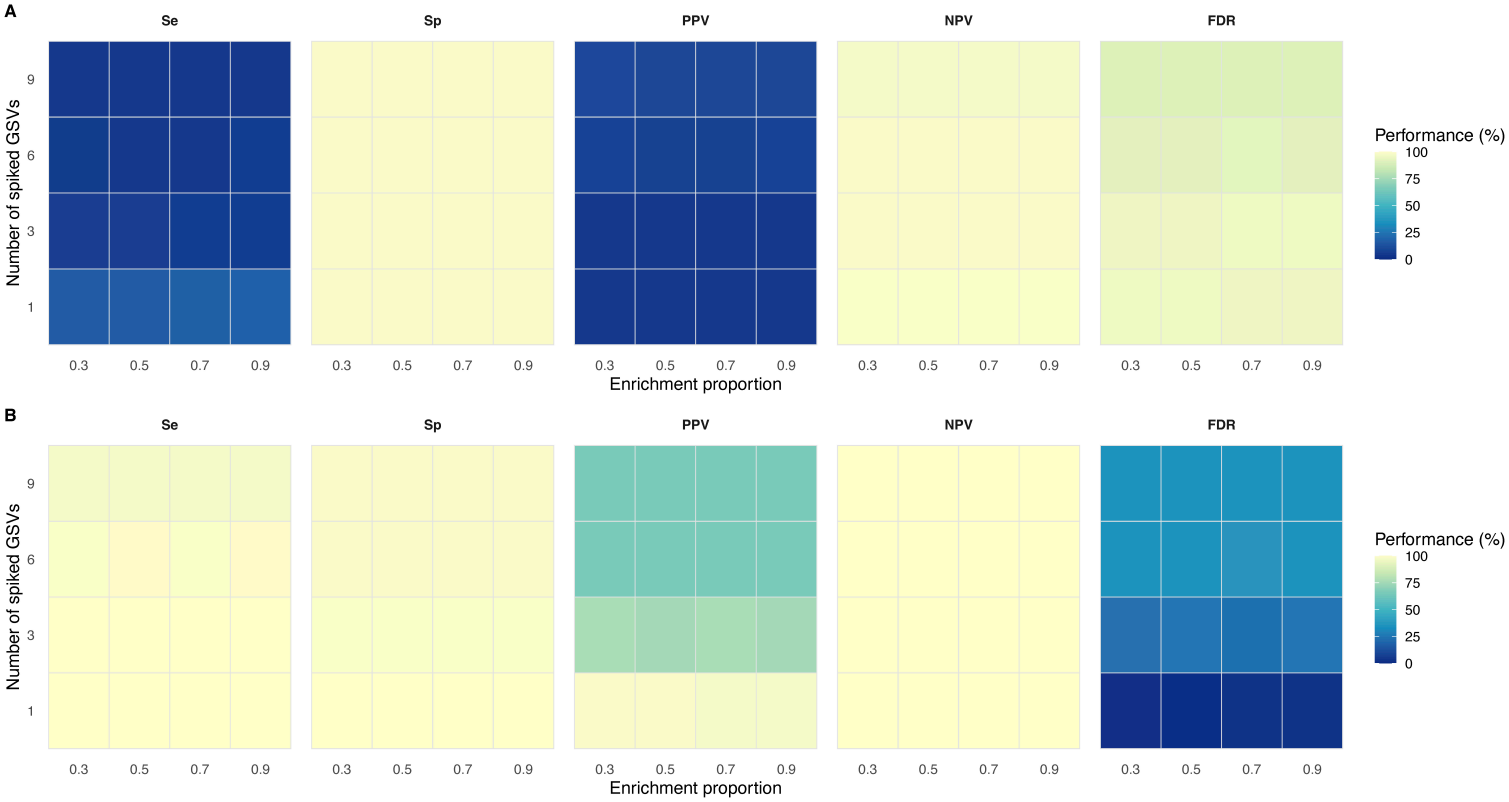
Performance of Kraken2 and Themisto/mSWEEP. Sensitivity (Se), specificity (Sp), positive predictive value (PPV), negative predictive value (NPV), and false discovery rate (FDR) obtained for Kraken2 **(A)** and Themisto/mSWEEP **(B)** per number of spiked GSVs and enrichment.

Overall mean Se, Sp, PPV, NPV, and FDR for Kraken2 classification was 8.5, 99.1, 6.8, 98.7, and 93.2%, respectively (SD = 21.5, 0.0, 12.0, 0.8, and 12.0%). Minimal variations were observed across enrichment proportions (Figure 3). Best performances of Se, Sp, PPV, NPV, and FDR were observed for a single GSV, and deteriorated with increasing numbers of spiked GSVs, except for PPV and FDR (Figure 3). For a single GSV, Se, Sp, PPV, NPV, and FDR were 17.3, 99.2, 4.7, 99.2, and 95.3%, respectively (SD = 37.8, 0.1, 10.4, 0.4, and 10.4%), compared to 4.7, 99.1, 10.0, 91.1, and 90.0%, respectively, for nine spiked GSVs (SD = 6.8, 0.0, 13.6, 0.6, and 13.6%) (Table 2).

**Table 2:** Descriptive statistics, including mean, median, standard deviation (SD), interquartile range (IQR) and total number of iterations (*N*) of the sensitivity (Se), specificity (Sp), positive predictive value (PPV), negative predictive value (NPV), and false discovery rate (FDR) per enrichment and the number of spiked GSVs for Kraken2 alignment.

**Performance metric**	***N***	**Mean**	**Median**	**Standard deviation**	**IQR**
***Se***					
**Enrichment**					
30%	4,000	8.5	0.0	21.5	0.0–11.1
50%	4,000	8.2	0.0	21.0	0.0–11.1
70%	4,000	8.5	0.0	21.6	0.0–11.1
90%	4,000	8.6	0.0	21.9	0.0–11.1
***N*** **of GSV's**					
1	4,000	17.3	0.0	37.8	0.0–0.0
3	4,000	6.3	0.0	13.8	0.0–0.0
6	4,000	5.6	0.0	9.1	0.0–16.7
9	4,000	4.7	0.0	6.8	0.0–11.1
***Sp***					
**Enrichment**					
30%	4,000	99.1	99.1	0.0	99.1–99.1
50%	4,000	99.1	99.1	0.0	99.1–99.1
70%	4,000	99.1	99.1	0.0	99.1–99.1
90%	4,000	99.1	99.1	0.0	99.1–99.1
***N*** **of GSV's**					
1	4,000	99.2	99.1	0.1	99.1–99.1
3	4,000	99.1	99.1	0.0	99.1–99.1
6	4,000	99.1	99.1	0.0	99.1–99.1
9	4,000	99.1	99.1	0.0	99.1–99.1
***PPV***					
**Enrichment**					
30%	4,000	6.9	0	12.0	0.0–25.0
50%	4,000	6.8	0	11.9	0.0–25.0
70%	4,000	6.8	0	11.9	0.0–25.0
90%	4,000	6.9	0	12.1	0.0–25.0
***N*** **of GSV's**					
1	4,000	4.7	0	10.4	0.0–0.0
3	4,000	4.7	0	10.0	0.0–0.0
6	4,000	8.0	0	12.5	0.0–25.0
9	4,000	10.0	0	13.6	0.0–25.0
***NPV***					
**Enrichment**					
30%	4,000	99.8	99.7	0.1	99.7–99.7
50%	4,000	99.2	99.1	0.1	99.1–99.1
70%	4,000	98.4	98.3	0.2	98.3–98.6
90%	4,000	97.5	97.4	0.2	97.4–97.7
***N*** **of GSV's**					
1	4,000	99.2	99.0	0.4	99.0–99.0
3	4,000	97.0	97.0	0.4	97.0–97.0
6	4,000	94.1	94.1	0.5	94.1–95.0
9	4,000	91.1	91.1	0.6	91.1–92.0
***FDR***					
**Enrichment**					
30%	4,000	93.1	100	12.0	75.0–100
50%	4,000	93.2	100	11.9	75.0–100
70%	4,000	93.2	100	11.9	75.0–100
90%	4,000	93.1	100	12.1	75.0–100
***N*** **of GSV's**					
1	4,000	95.3	100	10.4	100–100
3	4,000	95.3	100	10.0	100–100
6	4,000	92.0	100	12.5	75.0–100
9	4,000	90.0	100	13.6	75.0–100

### 3.4 Performance of Themisto/mSWEEP (≥1% relative abundance)

Average percentage of reads correctly classified as the spiked GSV(s) by Themisto/mSWEEP was 84.9% (SD = 25.0%), with a first quartile of 73.7% and a third quartile of 100%. Mean percentage of correctly classified reads did not vary across different enrichment proportions (lowest = 84.8%, highest = 85.0%), but varied based on the number of spiked GSVs (Table 1). The highest mean percentage of correctly classified reads was 98.8% (SD = 6.4%) for a single spiked GSV, whereas the lowest was 71.9% (SD = 9.4%) for iterations with 9 spiked GSVs (Table 3). For all enrichment proportions and numbers of spiked GSVs, the maximum percentage reached was 100%.

**Table 3:** Descriptive statistics, including mean, median, standard deviation (SD), interquartile range (IQR) and total number of iterations (*N*) of the sensitivity (Se), specificity (Sp), positive predictive value (PPV), negative predictive value (NPV), and false discovery rate (FDR) per enrichment and the number of spiked GSVs for Themisto/mSWEEP pseudo-alignment.

**Performance metric**	***N***	**Mean**	**Median**	**Standard deviation**	**IQR**
***Se***					
**Enrichment**					
30%	4,000	99.1	100	3.4	100–100
50%	4,000	99.1	100	3.4	100–100
70%	4,000	99.1	100	3.5	100–100
90%	4,000	99.2	100	3.3	100–100
***N*** **of GSV's**					
1	4,000	100.0	100.0	0.0	100–100
3	4,000	100.0	100.0	0.0	100–100
6	4,000	99.5	100.0	2.9	100–100
9	4,000	97.0	100.0	5.6	100–100
***Sp***					
**Enrichment**					
30%	4,000	99.3	99.4	0.7	98.8–100
50%	4,000	99.3	99.4	0.7	98.8–100
70%	4,000	99.3	99.4	0.7	98.8–100
90%	4,000	99.3	99.4	0.7	98.8–100
***N*** **of GSV's**					
1	4,000	100.0	100.0	0.1	100–100
3	4,000	99.6	99.7	0.4	99.4–100
6	4,000	99.0	99.1	0.4	98.8–99.4
9	4,000	98.6	98.5	0.5	98.2–98.8
***PPV***					
**Enrichment**					
30%	4,000	76.4	75.0	18.6	60.0–100
50%	4,000	75.8	75.0	18.7	60.0–100
70%	4,000	76.1	75.0	18.7	60.0–100
90%	4,000	75.8	75.0	18.6	60.0–100
***N*** **of GSV's**					
1	4,000	98.0	100	10.1	100–100
3	4,000	75.9	75.0	19.7	60.0–100
6	4,000	64.7	66.7	10.8	60.0–75.0
9	4,000	65.5	64.3	8.0	60.0–69.2
***NPV***					
**Enrichment**					
30%	4,000	100	100	0.1	100–100
50%	4,000	100	100	0.1	100–100
70%	4,000	100	100	0.1	100–100
90%	4,000	100	100	0.1	100–100
***N*** **of GSV's**					
1	4,000	100	100	0.0	100–100
3	4,000	100	100	0.0	100–100
6	4,000	100	100	0.1	100–100
9	4,000	99.9	100	0.1	100–100
***FDR***					
**Enrichment**					
30%	4,000	23.6	25.0	18.6	0.0–40.0
50%	4,000	24.2	25.0	18.7	0.0–40.0
70%	4,000	23.9	25.0	18.7	0.0–40.0
90%	4,000	24.2	25.0	18.6	0.0–40.0
***N*** **of GSV's**					
1	4,000	2.0	0.0	10.1	0.0–0.0
3	4,000	24.1	25.0	19.7	0.0–40.0
6	4,000	33.3	33.3	10.8	25.0–40.0
9	4,000	35.7	35.7	8.0	30.8–40.0

Overall mean Se, Sp, PPV, NPV, and FDR for Themisto/mSWEEP classification was 99.1, 99.3, 76.0, 99.9, and 24.0%, respectively (SD = 3.4, 0.7, 18.7, 0.1, and 18.7%). Minimal variations were observed across enrichment proportions (Figure 3). Best performances of Se, Sp, PPV, NPV, and FDR were observed for a single spiked GSV and deteriorated with increasing numbers of spiked GSVs (Figure 3). For a single GSV, Se, Sp, PPV, NPV, and FDR were 100, 100, 98.0, 100, and 2%, respectively (SD = 0.0, 0.1, 10.1, 0.0, and 10.1%), compared to 97.0, 98.6, 65.5, 99.9, and 35.7%, respectively, for 9 spiked GSVs (SD = 5.6, 0.5, 8.0, 0.1, and 8.0%) (Table 3).

### 3.5 Performance of targeted enrichment

A model incorporating three-way interactions failed to converge for Se and Sp. As a result, only two-way interactions between classification method and enrichment, and classification method and number of spiked GSVs were tested for these metrics.

For Se, there was no interaction between classification method and enrichment (*P* = 0.92), leading to removal of this term. There was a significant interaction between classification method and number of spiked GSVs (*P* < 0.001). Across all levels of spiked GSVs, Se was higher for mSWEEP/Themisto than Kraken2 (Table 4; *P* < 0.001 for all comparisons). Within each classification method, Se decreased as the number of spiked GSVs increased from 1 to 9 (*P* < 0.001 for all pairwise comparisons, except for 1 vs. 3 spiked GSVs in mSWEEP/Themisto). For Kraken2, Se declined from 17.5 to 4.7%. For mSWEEP, Se remained 100% for 1 and 3 spiked GSVs, then declined to 99.4 and 96.9% for 6 and 9 GSVs, respectively (Table 4). Enrichment as a main effect was not significant (*P* = 0.77), but classification method and number of spiked GSVs were (*P* < 0.001; *P* < 0.001, respectively).

**Table 4:** Effect of the classification method, the number of spiked GSVs, and the enrichment proportion on the performance metrics.

**Performance metric**	**Kraken2**			**Themisto/mSWEEP**		
	**95% CI**			**95% CI**		
	**Margin**	**Lower**	**Upper**	**Margin**	**Lower**	**Upper**
**Sensitivity**						
*N* of GSVs						
1	17.3	16.1	18.4	100^a^	NE	NE
3	6.3	5.9	6.7	100^a^	NE	NE
6	5.6	5.3	5.9	99.5	99.4	99.6
9	4.7	4.5	4.9	97.0	96.9	97.2
**Specificity**						
*N* of GSVs						
1	99.2	99.1	99.2	100	100	100
3	99.1^b^	99.1	99.2	99.6	99.6	99.6
6	99.1^b^	99.1	99.1	99.0	99.0	99.0
9	99.1^b^	99.1	99.1	98.6	98.6	98.6
**Positive predictive value**						
*N* of GSVs						
1	5.6^c^	6.2	6.0	95.8	95.2	96.4
3	5.9^c^	5.5	6.3	70.4	69.7	71.1
6	10.0	9.5	10.5	63.0	62.5	63.5
9	12.4	11.9	13.0	64.6	64.2	65.0
**Negative predictive value**						
*N* of GSVs						
1	99.8	99.8	99.8	100^a^	NE	NE
3	99.2	99.2	99.2	100^a^	NE	NE
6	98.4	98.3	98.4	100	100	100
9	97.5	97.5	97.6	99.9	99.9	99.9
**False discovery rate**						
*N* of GSVs						
1	94.4^c^	94.0	94.8	4.2	3.4	4.8
3	94.1^c^	93.7	94.5	29.6	28.9	30.2
6	90.0	89.5	90.5	37.0	36.5	37.5
9	87.6	87.0	88.1	35.4	35.0	35.8

Values are predictive margins transformed as percentages.

^a^The corresponding observations were dropped from the model due to perfect prediction. Therefore, the margin and standard error are non-estimable (NE). Here, a value of 100 for the margin is shown in the table to highlight the perfect prediction.

^b^Not statistically different (*P* > 0.05).

^c^Not statistically different (*P* = 0.226).

Similarly, there was no interaction between the classification method and enrichment (*P* = 0.7354) for Sp, which led to the removal of this term. There was a significant interaction between classification method and number of spiked GSVs (*P* < 0.001). Specificity was higher for mSWEEP/Themisto than Kraken2 when 1 or 3 GSVs were spiked, with the opposite trend when 6 or 9 GSVs were spiked (Table 4; *P* < 0.001 for all comparisons). For Kraken2, Sp declined from 99.2 to 99.1% from 1 to 3 spiked GSV (*P* < 0.05), and remained at 99.1% throughout. For mSWEEP, Sp declined. From 100 (1 spiked GSV) to 98.6% (9 spiked GSVs) (Table 4; *P* < 0.001 for all comparisons). Enrichment as a main effect was not significant (*P* = 0.7935), but classification method and number of spiked GSVs were (*P* < 0.001; *P* < 0.001, respectively).

For PPV, the three-way interaction was not significant (*P* = 0.81) and removed from the model, as was the interaction between classification method and enrichment proportion (*P* = 0.98). There was a significant interaction between classification method and number of spiked GSVs (*P* < 0.001). Across all numbers of spiked GSVs, PPV was higher for mSWEEP/Themisto than for Kraken2 (Table 4; *P* < 0.001 for all pairwise comparisons). For Kraken2, PPV increased from 5.6% for 1 and 3 spiked GSVs to 10.0 and 12.4% for 6 and 9 spiked GSVs, respectively (*P* < 0.001 for all pairwise comparisons, except for 1 vs. 3 spiked GSVs; *P* = 0.23). For mSWEEP/Themisto, PPV decreased from 95.8% for 1 spiked GSV to 70.4, 63.0, and 64.6% for 3, 6, and 9 spiked GSVs, respectively (Table 4; *P* < 0.001 for all pairwise comparisons). Enrichment as a main effect was not significant (*P* = 0.60), but classification method and number of spiked GSVs were (*P* < 0.001; *P* < 0.001, respectively).

For NPV, the three-way interaction model was plagued with collinearity. Therefore, a two-way interaction model was evaluated. The interaction term between enrichment and classification method was not significant (*P* = 0.8184) and removed from the model. There was a significant interaction between classification method and number of spiked GSVs (*P* < 0.001) with perfect prediction in two levels of this term. For Kraken2, NPV significantly decreased from 99.8% for 1 spiked GSVs to 97.5% for 9 spiked GSVs (*P* < 0.001 for all pairwise comparisons). For mSWEEP/Themisto, NPV was not estimable for one and three spiked GSV (due to perfect prediction), decreasing from 100 to 99.9% for 6 and 9 spiked GSV, respectively (Table 4; *P* < 0.001 for all pairwise comparisons). Enrichment as a main effect was not significant (*P* = 0.9939), but classification method and number of spiked GSVs were (*P* < 0.001; *P* < 0.001, respectively).

For FDR, the three-way interaction was non-significant (*P* = 0.80) and removed from the model. Similarly, there was no interaction between classification method and enrichment proportion (*P* = 0.98) and was excluded. A significant interaction between classification method and number of spiked GSVs was detected (*P* < 0.001). Across all numbers of spiked GSVs, FDR was lower for mSWEEP/Themisto than Kraken2 (Table 4; *P* < 0.001 for all pairwise comparisons). For Kraken2, FDR declined from 94.4% for 1 spiked GSV, to 94.1, 90.0 and 87.6% for 3, 6 and 9 spiked GSVs, respectively (*P* < 0.001 for all pairwise comparisons, except for 1 vs. 3 spiked GSVs; *P* = 0.23). For mSWEEP/Themisto, FDR increased from 4.2% for 1 spiked GSV, to 28.9, 36.5, and 35.8% for 3, 6 and 9 spiked GSVs, respectively (Table 4; *P* < 0.001 for all pairwise comparisons). Enrichment as a main effect was not significant (*P* = 0.60), but classification method and number of spiked GSVs were (*P* < 0.001; *P* < 0.001, respectively).

### 3.6 Performance of Kraken2 and Themisto/mSWEEP with different thresholds

In this sensitivity analysis, increasing the threshold resulted in a decrease in sensitivity. However, the improvement in specificity led to a lower proportion of false positives, which in turn increased the PPV and reduced the FDR, as the prevalence of GSVs remained constant (Supplementary material).

### 3.7 Sensitivity analysis

In order to assess the impact of simulating sequencing error, we compared the findings for Kraken2 and Themisto/mSWEEP from the main simulation with those from the sensitivity analysis with the 31 minimizer length/k-mer length value. We observed similar trends with values very close numerically for Kraken2 and Themisto/mSWEEP for all metrics (Supplementary Figure 2), except for PPV which was higher than in the main simulation for Kraken2 (20.9, 21.5, 31.7, and 33.9% for 1, 3, 6, and 9 spiked GSVs, respectively; Supplementary Figure 2). Correspondingly, FDR values were lower in the sensitivity analysis than in the main simulation for Kraken2 (79.1, 78.5, 68.3, 66.1% for 1, 3, 6, and 9 spiked GSVs, respectively) (Supplementary Figure 2). The effect of minimizer length/k-mer length on the performance metrics did not significantly vary by classification method or the number of spiked GSVs and was only statistically significant on Se and Sp, in which using a minimizer length/k-mer length of 20 resulted in lower or higher values, respectively, compared to 25 and 31 k-mers, after adjusting for classification method and the number of spiked GSVs (*P* < 0.005).

## 4 Discussion

An *in-silico* evaluation of the suitability of a TE shotgun sequencing approach for detecting and classifying *M. bovis* strains (as GSVs) in metagenomic samples from milk was undertaken. The evaluation included a combination of multiple metagenomic bioinformatic tools, including MashTree, TreeCluster, Kraken2, and Themisto/mSWEEP, and various key parameters such as number of spiked GSVs and enrichment proportions. The Themisto/mSWEEP tool consistently outperformed Kraken2, achieving a higher percentage of reads correctly classified as the spiked GSVs. Kraken2's performance was suboptimal across all enrichment proportions and number of spiked GSVs, whereas Themisto/mSWEEP maintained high accuracy with only a slight decline in performance as more GSVs were added to the simulation.

Enrichment proportions did not impact the performance metrics, indicating that the enrichment of *M. bovis* DNA to at least 30% of the sequenced reads is enough to obtain robust GSV-level data under the conditions of this simulation. However, simulations involve controlled and idealized conditions, such as known number of reads, uniform strain distribution, and optimal sample preparation, which minimizes noise and variability. These conditions may not fully reflect the complexity of actual samples, where challenges such as uneven coverage, low-abundance strains, and sample variability persist (42).

Empirical data suggest that targeted enrichment can still provide important insights even in the presence of low abundant strains (18, 43). While higher enrichment levels generally increase sensitivity, they are associated with higher costs and may be unattainable in certain sample matrices as the enrichment depends upon the bait design, the chemistry, and the abundance of the target DNA in the sample (18, 42, 44).

The observed decrease in precision with the spiking of more GSVs in some models could be attributed to increased sample variability. More GSVs could create overlapping signals, complicating accurate identification and assignment of reads to the correct GSV. As a result, the probability of FP and FN increases, adversely affecting diagnostic test performance indicators. Nevertheless, even with 9 GSVs, Themisto/mSWEEP successfully classified 72% of reads to the correct GSV with a Se of 97%. Kraken2 increased PPV performance with more spiked GSVs could be due to the increased genomic diversity and improved alignment opportunities, allowing it to match reads more accurately to its reference database. However, this improvement may reach a limit if too many GSVs are introduced due to overlapping sequences.

The results from this simulation suggest that pseudo-alignment-based tools may be more suitable to handle the data obtained via targeted enriched metagenomics. Kraken2 does not rely on traditional alignment methods but instead uses a k-mer-based classification approach that rapidly processes sequences by fragmenting them into smaller k-mer segments (23). Unlike BLAST, which compares sequences through pairwise base-to-base alignments (45), Kraken2 uses exact k-mer matches, which may be too rigid for applications where subtle genetic variations exist between closely related strains. Pseudo-alignment methods like Themisto/mSWEEP, on the other hand, focus on capturing broader similarities between genomes without requiring perfect base-to-base matches, making them more adaptable to highly diverse genomic data (46). Although BLAST is accurate for detailed alignments, it is computationally intensive, making it less suitable for analyzing larger datasets. Kraken2's emphasis on exact k-mer matching could miss minor variations between closely related strains, whereas pseudo-alignment tools like Themisto/mSWEEP are better equipped handling this due to their focus on broader genomic relationships, as the idea of pseudoalignment is based on the fact that the sufficient statistics of the quantification of variants are the assignments of reads to indexes, not their alignment (46, 47). This characteristic may explain why Themisto/mSWEEP outperformed Kraken2 in our study. Additionally, k-mer length is a critical parameter for both tools. Of note, Themisto uses a default k-mer length of 31 base pairs and indexes all k-mers. By contrast, Kraken2 uses minimizers of the k-mers for indexing (defaults are 31-bp minimizers and k-mers of length 35). Therefore, the default length of the actual k-mers used for indexing is the same in both tools. Additionally, our sensitivity analysis did not find a significant effect of lower values of k-mer length in most of the performance metrics. However, future studies should assess the effect of higher k-mer length values on the performance of Themisto/mSWEEP, as this value was only lowered from the default to maintain comparability with Kraken2.

Traditionally, the term “strain” has referred to a pure culture or isolate within a culture-based approach (48). However, as research increasingly shifted toward culture-free methods, a widely accepted definition for “strain” in this context is still absent (49). The lack of consensus has led to inconsistencies in terminology and has hindered effective communication among researchers (26). Consequently, this study defined “strain” as a genomically clustered sequencing variant (GSV), providing a more explicit definition of “strain diversity”, and enhancing clarity in our analysis and research findings.

Although the literature on mixed *Mycoplasma* infections in cattle has primarily focused on mixed species or mixed infections with other pathogens (1, 50, 51), the concept of mixed strain infections is becoming increasingly relevant for both animal and human pathogens (26). Existing methods for strain identification have been documented in literature (52–54). However, most of these methods are bound by several limitations. For example, SplitStrain (53) is limited to identifying either a single (pure) strain or a mixed-strain DNA sample involving 2 strains. MixInfect (54) estimates the ratio of heterozygous calls to total SNPs, using a threshold to identify mixed samples. While this approach can estimate mixture proportions, it lacks the capability to resolve individual strains. Mixed Infection Estimator (52), originally developed for *Clostridioides difficile*, depends on a custom sequence database and would only obtain mixture proportion estimation in our context. Consequently, combining targeted enriched metagenomics with Themisto/mSWEEP offers a robust approach to explore within-species strain diversity in *M. bovis* and other pathogens.

### 4.1 Limitations and future directions

Although Themisto/mSWEEP successfully identified the correct spiked GSVs in this study, and the GSV classification moderately agrees with the MLSTs, the biological relevance of GSVs remains unclear. Another limitation is the lack of experimental data in this study. The simulations were conducted under tightly controlled and idealized conditions that cannot fully mimic the biological and technical complexity of real metagenomic samples. Several important factors were not simulated, such as host DNA contamination, milk microbiota, variability in DNA extraction and enrichment efficiencies, as well as biases during library preparation and sequencing. Therefore, the performance observed in this study likely represents upper-bound estimates that may not mirror the performance in clinical settings. Additionally, the observed independence of TE performance from the enrichment proportion should be interpreted as conditional on the assumptions of the simulation.

The next steps include conducting a field study using real milk samples to validate the *in-silico* results. Following this, analyzing samples collected from cows with different clinical signs, subclinical carriers, and from various locations of the body using the validated protocol should provide deeper insights into the relevance of these GSVs. We believe the proposed approach in this study has significant potential to ultimately help clarify the relationship between *M. bovis* strains and disease presentation, treatment plans, and transmission dynamics on dairy farms.

## 5 Conclusion

This study demonstrated that *in silico* clustering of WGS data based on GSVs using Themisto/mSWEEP successfully identified up to 9 spiked GSVs. Se, Sp, and PPV remained high for both single and mixed-strain DNA samples involving 9 GSVs. In contrast, Kraken2 performed poorly as a classification method, with significantly low Se and PPV, and higher FDR, making it an unsuitable method for target enrichment methodologies. Finally, enrichment proportions did not impact the performance metrics, suggesting that the enrichment of *M. bovis* DNA to at least 30% of the sequenced reads is enough to obtain robust GSV-level data.

## Data Availability

The scripts used in this study can be accessed at https://github.com/robertvaleris/Mycoplasma_bovis_TE_simulation.
