# Supplementary material for: In silico performance of a targeted enriched metagenomics approach to infer Mycoplasma bovis strains in milk
Source: Front Vet Sci. 2026 Mar 18;13:1770245. doi: 10.3389/fvets.2026.1770245 (PMC13041548; doi:10.3389/fvets.2026.1770245)
Supplement: Supplementary file 1 [file Data_Sheet_1.pdf]

Supplementary Material

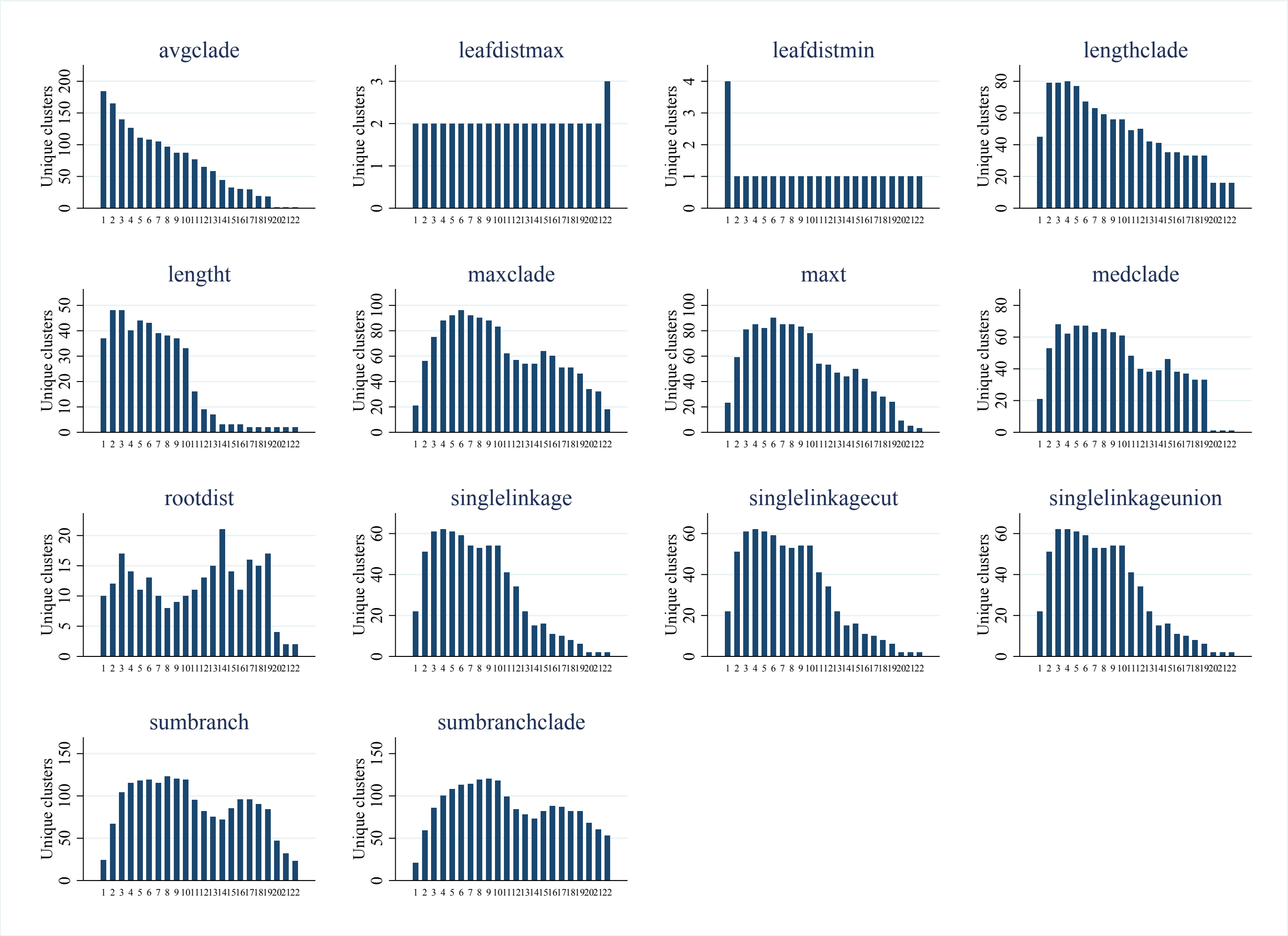

Supplementary Figure 1. Determination of the unique genomic clusters in 620 *Mycoplasma bovis* whole genome sequences in TreeCluster. The evaluated methods (n=14) include average clade (avgclade), maximum leaf distance (leafdistmax), minimum leaf distance (leafdistmin), length (lengtht), length of the clade (lengthclade), maximum (maxt), maximum clade (maxclade), median clade (medclade), root distance (rootdist), single linkage (singlelinkage), single linkage using the Metin's cut algorithm (singlelinkagecut), single linkage using the Niema's union algorithm (singlelinkageunion), sum of the branch (sumbranch), and sum of the branch of the clade (sumbranchclade). For each method, the following thresholds (n=22) were evaluated: 0.0001 (1), 0.0002 (2), 0.0003 (3), 0.0004 (4), 0.0005 (5), 0.0006 (6), 0.0007 (7), 0.0008 (8), 0.0009 (9), 0.001 (10), 0.002 (11), 0.003 (12), 0.004 (13), 0.005 (14), 0.006 (15), 0.007 (16), 0.008 (17), 0.009 (18), 0.01 (19), 0.2 (20), 0.3 (21), and 0.4 (22).

1 Sensitivity Analysis on the Effect of the Threshold for Classification

A sensitivity analysis was conducted with the objective of assessing the effect of the threshold on the performance of the classification tools. The threshold used (GSVs with ≥ 1% relative abundance) was increased to ≥ 5% and ≥ 10%. Our hypothesis was that increasing the threshold would increase the Sp and decrease Se. We were interested in assessing the impact on PPV, NPV, and FDR.

1.1 ≥ 5% of reads classified as the specific GSV (Kraken2) or ≥ 5% relative abundance (Themisto/mSWEEP)

1.1.1 Performance of Kraken2 with a custom database

Overall mean Se, Sp, PPV, NPV and FDR for Kraken2 classification was 2.1, 99.4, 1.3, 98.7, and 98.7%, respectively (SD = 13.0, 0.0, 6.4, 0.9, and 6.4%). Minimal variations were observed across enrichment proportions. Best performances of Se, Sp, PPV, NPV and FDR were observed for a single GSV, and deteriorated with increasing numbers of spiked GSVs, except for PPV and FDR. For a single GSV, Se, Sp, PPV, NPV and FDR were 6.4, 99.4, 2.1, 99.7, and 97.9%, respectively (SD = 24.5, 0.0, 8.2, 0.1 and 8.2%), compared to 0.0, 99.4, 0.0, 97.4, and 100%, respectively, for 9 spiked GSVs (SD = 0.0, 0.0, 0.0, 0.0, and 0.0%).

1.1.2 Performance of Themisto/mSWEEP

Overall mean Se, Sp, PPV, NPV and FDR for Themisto/mSWEEP classification was 90.6, 99.7, 83.1, 99.8, and 16.9%, respectively (SD = 14.4, 0.4, 15.8, 0.4, and 15.8%). Minimal variations were observed across enrichment proportions. Best performances of Se, Sp, PPV, NPV and FDR were observed for a single spiked GSV and deteriorated with increasing numbers of spiked GSVs. For a single GSV, Se, Sp, PPV, NPV and FDR were 100, 100, 98.0, 100, and 2%, respectively (SD = 0.0, 0.1, 9.8, 0.0, and 9.8%), compared to 72.6, 99.3, 73.4, 99.3, and 26.6%, respectively, for 9 spiked GSVs (SD = 13.4, 0.3, 11.2, 0.3, and 11.2%).

1.2 ≥ 10% of reads classified as the specific GSV (Kraken2) or ≥ 10% relative abundance (Themisto/mSWEEP)

1.2.1 Performance of Kraken2 with a custom database

Overall mean Se, Sp, PPV, NPV and FDR for Kraken2 classification was 1.3, 99.4, 0.6, 98.6, and 99.4%, respectively (SD = 10.8, 0.0, 4.7, 0.9, and 4.5%). Minimal variations were observed across

enrichment proportions. Best performances of Se, Sp, PPV, NPV and FDR were observed for a single GSV, and deteriorated with increasing numbers of spiked GSVs, except for PPV and FDR. For a single GSV, Se, Sp, PPV, NPV and FDR were 4.4, 99.4, 1.5, 99.7, and 98.5%, respectively (SD = 20.6, 0.0, 6.9, 0.1 and 6.9%), compared to 0.0, 99.4, 0.0, 97.4, and 100%, respectively, for 9 spiked GSVs (SD = 0.0, 0.0, 0.0, 0.0, and 0.0%).

1.2.2 Performance of Themisto/mSWEEP

Overall mean Se, Sp, PPV, NPV and FDR for Themisto/mSWEEP classification was 75.0, 100, 89.9, 99.4, and 10.2%, respectively (SD = 30.2, 0.2, 16.0, 0.8, and 16.0%). Minimal variations were observed across enrichment proportions. Best performances of Se, Sp, PPV, NPV and FDR were observed for a single spiked GSV and deteriorated with increasing numbers of spiked GSVs. For a single GSV, Se, Sp, PPV, NPV and FDR were 100, 100, 98.1, 100, and 1.9%, respectively (SD = 0.0, 0.1, 9.7, 0.0, and 9.7%), compared to 30.4, 99.8, 80.7, 98.2, and 19.3%, respectively, for 9 spiked GSVs (SD = 11.9, 0.2, 20.4, 0.3, and 20.4%).

1.2.3 Summary

Increasing the threshold deteriorated the performance for Kraken2, as Se and PPV dropped considerably. By contrast, a different trend was observed in Themisto/mSWEEP: with higher thresholds, Sp and PPV increased while the decrease in Se was acceptable.

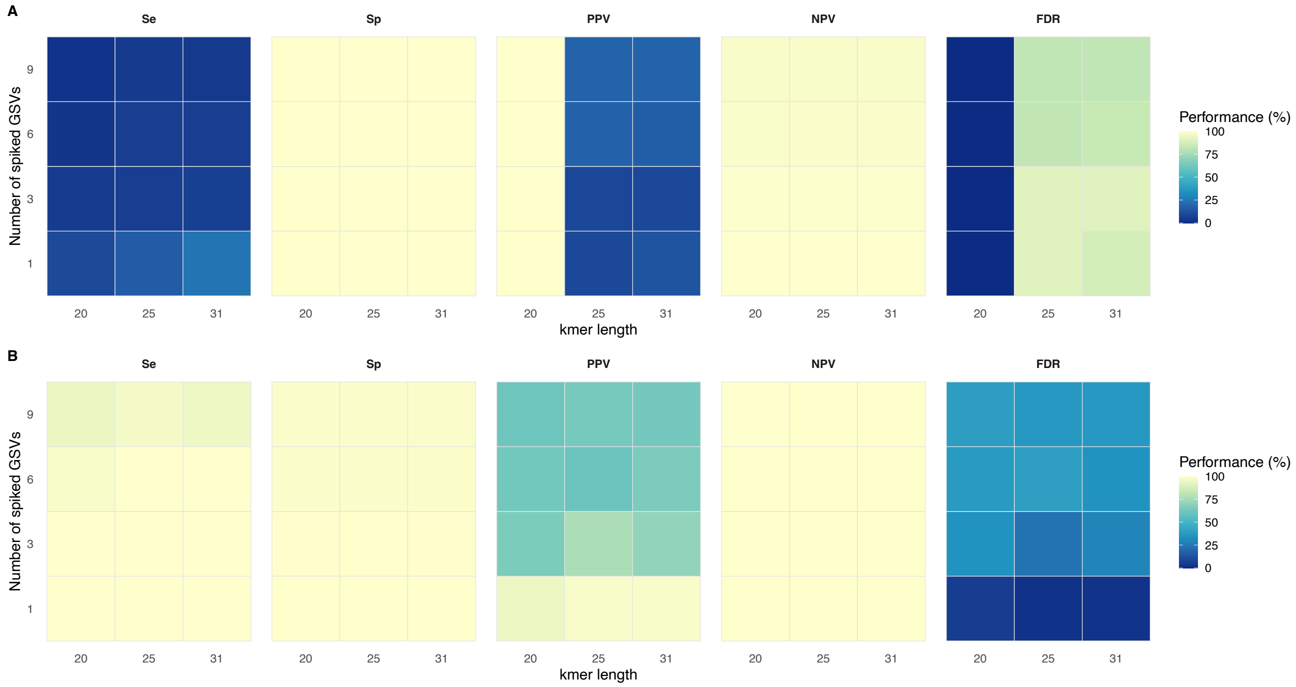

Supplementary Figure 2. Performance of Kraken2 and Themisto/mSWEEP during the sensitivity analysis on the effect of k-mer length. Sensitivity (Se), Specificity (Sp), Positive Predictive Value (PPV), Negative Predictive Value (NPV) and False Discovery rate (FDR) obtained for Kraken2 (A) and Themisto/mSWEEP (B) per number of spiked GSVs and k-mer length.
